# Blood flow pattern in eye before development of type 3 macular neovascularization

**DOI:** 10.1371/journal.pone.0283202

**Published:** 2023-03-16

**Authors:** Saya Yamaguchi, Ichiro Maruko, Ruka Maruko, Taiji Hasegawa, Tomohiro Iida

**Affiliations:** Department of Ophthalmology, Tokyo Women’s Medical University School of Medicine, Shinjuku, Tokyo, Japan; Kobe University Graduate School of Medicine School of Medicine: Kobe Daigaku Daigakuin Igakukei Kenkyuka Igakubu, JAPAN

## Abstract

**Purpose:**

To determine the blood flow pattern of eyes before the development of type 3 macular neovascularization (MNV) by optical coherence tomography angiography (OCTA).

**Study design:**

Retrospective study.

**Subjects:**

Ten eyes of 10 patients (4 men and 6 women, mean age 80.4 years) diagnosed with unilateral Type 3 MNV who developed type 3 MNV in the fellow normal eye during the follow-up period were studied.

**Methods:**

The time of onset of type 3 MNV was defined as the time when retinal exudation was detected by OCT. The blood flow of a 3 x 3 mm or 6 x 6 mm area in the deep capillary plexus (DCP) and the outer retina (OR) including the central fovea were assessed at the onset and at 6 months prior to the onset of the type 3 MNV.

**Results:**

All MNVs that developed in the fellow eye were type 3 MNVs. Abnormal blood flow signals in the MNVs were detected in the DCP and/or the OR by OCTA at the onset in all cases. Eight of the 10 eyes had OCTA recordings prior to the development of the MNV: 3 eyes had non-exudative MNVs only in the DCP and 5 eyes had non-exudative MNVs in the DCP and OR. The exudation appeared on the average 3.5 months after the non-exudative MNV was observed in the fellow eyes.

**Conclusions:**

A non-exudative MNV in the fellow eyes can already be observed by OCTA in eyes before the onset of the exudation. Knowing this will help clinicians not only how to treat these eyes appropriately but will also help in determining the origin of the MNV.

## Introduction

A type 3 macular neovascularization (MNV), also known as retinal angiomatous proliferation (RAP), is one of the subtypes of age-related macular degeneration (AMD) with an MNV [1–3]. It is usually bilateral, and in unilateral cases, the type 3 MNV develops in the fellow eye within 3 years [4]. Although the angiogenesis of type 3 MNV occurs within the retina, it is difficult to detect it in the nascent stage because patients are rarely examined because they have no symptoms at this early stage. Sacconi et al [5] reported that optical coherence tomography angiography (OCTA) could detect the non-exudative type 3 MNV in the deep capillary plexus (DCP) and outer retina (OR) even in the absence of exudation by OCTA. However, there are no details about the early angiogenesis pattern prior to exudation in patients with type 3 MNV.

Thus, the purpose of this study was to determine the early angiogenic pattern of eyes with type 3 MNV before the development of the exudations. To accomplish this, we followed the fellow normal eyes of patients with unilateral type 3 MNV with OCTA. Our findings showed that abnormal blood vessel patterns were present even before the exudation was detected.

## Methods

The procedures used in this retrospective study conformed to the tenets of the Declaration of Helsinki and were approved by the Institutional Review Board of Tokyo Women’s Medical University. All patients signed a written informed consent before beginning the treatments.

There were 47 patients who were diagnosed with unilateral type 3 MNV since January 2006 in the Department of Ophthalmology, Tokyo Women’s Medical University Hospital. OCTA examinations were performed on both the affected and normal fellow eyes in this group since 2016 when OCTA examinations were begun. Ten of the normal fellow eyes developed type 3 MNV during the follow-up period. Because both eyes of all of these eyes had been examined by OCTA, we were able to examine the blood flow pattern at the site of the newly developed MNV before it developed.

All patients underwent fundus examination by slit-lamp biomicroscopy, color fundus photography, optical coherence tomography (OCT), and OCTA. All eyes were examined by swept-source OCT (DRI-OCT, Topcon, Japan) and OCTA (RTVue XR Avanti, Optovue, Fremont, CA). The eyes were diagnosed with unilateral type 3 MNV when subretinal, intraretinal, or preretinal hemorrhages, retinal edema, and retina–retina or retina–choroid blood vessel anastomosis were present. Nine of the ten originally affected eyes was diagnosed to have a type 3 MNV by fluorescein angiography (FA) and indocyanine green angiography (IA). The remaining 1 patient did not undergo angiography due to allergy to the contrast dye, and the diagnosis was made based on other clinical results.

We determined the vasogenic stage of the type 3 MNV or RAP according to the classification of Yannuzzi and associates [2]: stage 1, intraretinal neovascularization; stage 2A, subretinal neovascularization; stage 2B, subretinal neovascularization with pigment epithelial detachment (PED); and stage 3, neovascularization derived from retinal vessels and anastomosis with choroidal vessels.

SS-OCT was performed with horizontal and vertical scans through the central fovea, and OCTA was performed with a 3 x 3 mm or 6 x 6 mm area including the foveal area. The onset of the type 3 MNV in the fellow eye was defined as the time of appearance of the retinal exudation in the OCT or OCTA image. The pattern of the blood flow in the DCP and OR was analyzed at the time of onset of the disease. The prior OCTA images were available for 6 months before the appearance of the exudation. These images were evaluated for the presence of abnormal blood flow signs at the site at the onset. We also determined the time from the identification of the abnormal blood flow signs to the appearance of the exudation.

## Results

Ten eyes of 10 patients (4 men and 6 women) were studied. All were Japanese with a mean age of 80.4 years, a median of 82 years, and a range of 76 to 90 years. At the time of the initial examination, all patients had soft drusen in both eyes. Three eyes were at stage 1, one was at stage 2A, three were stage 2B, and three were at stage 3 RAP (type 3 MNV) in the affected eyes by the Yannuzzi classification. All patients began treatment with intravitreal injection of aflibercept into the affected eye after the diagnosis.

None of the patients had retinal exudation or treatment in the fellow eyes at the time of the initial examination. At the onset of the exudation and diagnosis of AMD in the fellow eyes, the mean decimal best-corrected visual acuity (BCVA) was 1.02 (0.01 logMAR units) with a range 0.7 to 1.2.

Abnormal blood flow pattern indicating a type 3 MNV was confirmed to be present in both the DCP and the OR in all cases at the onset of exudation in the fellow eye. Prior OCTA images were available in 8 of the 10 eyes, and they were recorded within 6 months before the onset of the exudation. Examination of the site of the subsequent MNV showed abnormal blood flow pattern suggestive of non-exudative type 3 MNV in both the DCP and the OR in 2 eyes (Cases 1 and 5) and only in the DCP in 3 eyes (Cases 2, 3, and 6). No abnormal blood flow patterns were observed in the remaining three eyes. The average time from the detection of the nonexudative MNV to the onset of exudation was 3.5 months with a range of 2 to 6 months.

We present more detailed findings in 2 of the cases, and more detailed data for both eyes of all cases in Table 1.

**Table 1:** Characteristics of eyes with prior diagnosed and fellow eyes of all patients.

Case	Age	Sex	Fellow eye								Prior diagnosed eye		
			Eye	Visual acuity		flow signal of MNV					Stage	Visual acuity	
				(decimal)	(logMAR)	Before exudation		Onset exudation		Onset (M)		(decimal)	(logMAR)
						DCP	OR	DCP	OR				
1	75	M	OD	1.2	-0.08	+	+	+	+	5	2B	0.7	0.15
2	77	F	OD	1.0	0.00	+	−	+	+	3	1	1.0	0.00
3	78	F	OD	1.0	0.00	+	−	+	+	3	3	0.5	0.30
4	78	F	OD	1.0	0.00	−	−	+	+	3	1	1.2	-0.08
5	79	F	OD	1.2	-0.08	+	+	+	+	3	3	0.2	0.70
6	88	F	OS	1.2	-0.08	+	−	+	+	1	2B	0.5	0.30
7	90	M	OD	0.7	0.15	NA	NA	+	+	NA	1	1.2	-0.08
8	82	M	OD	0.8	0.10	NA	NA	+	+	NA	3	0.1	1.05
9	81	M	OD	1.2	-0.08	−	−	+	+	2	2A	0.5	0.30
10	76	F	OD	1.0	0.00	−	−	+	+	5	2B	0.2	0.70
Mean	80.4	M:F = 4:6		1.02	-0.01	(+) 5	(+) 2	(+) 10	(+) 10	3.1		0.46	0.33

DCP = deep capillary plexus

OR = outer retina

Case 3 (Figs 1–3)

**Fig 1:**
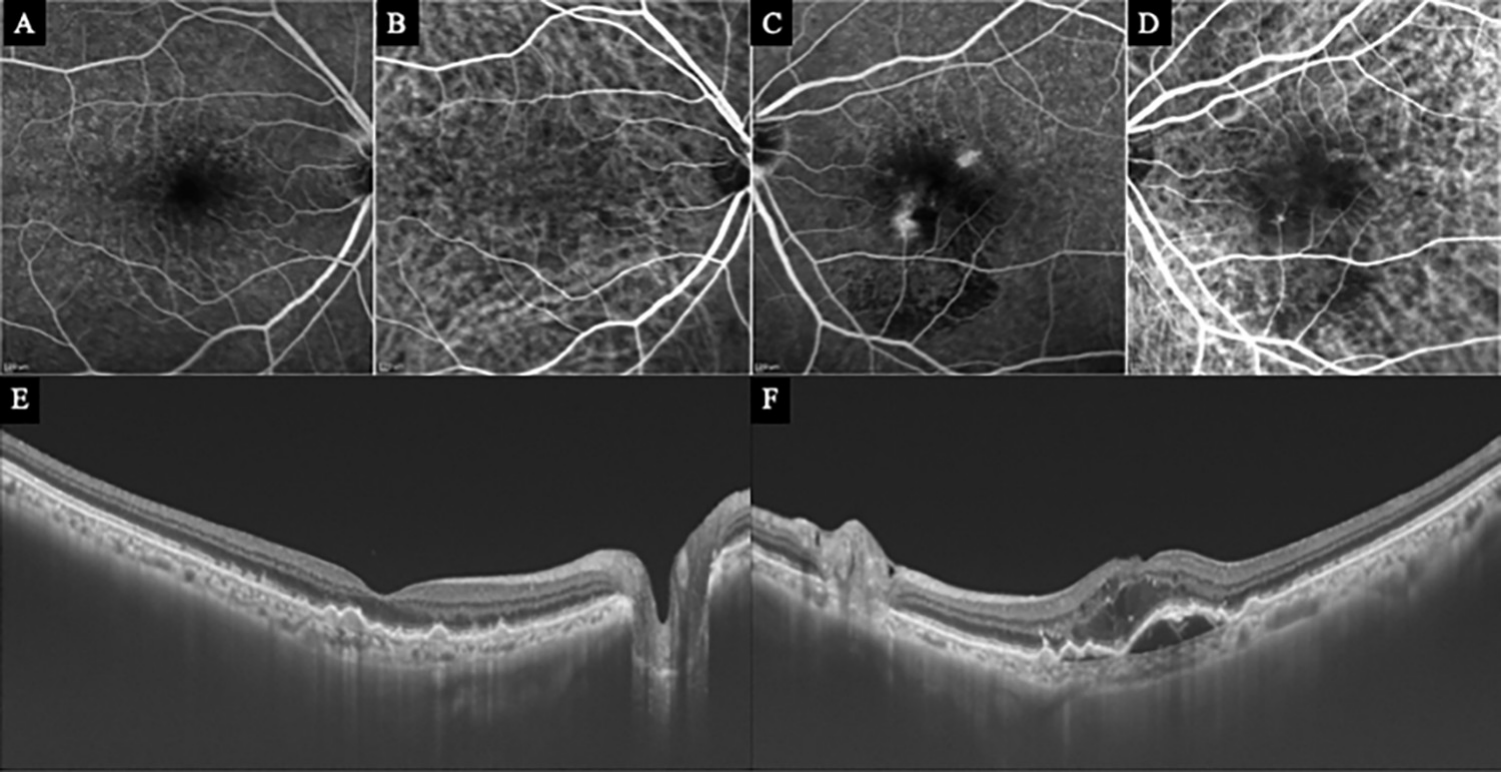
Fluorescein angiographic (FA), Indocyanine angiographic (ICGA), and optical coherence tomographic (OCT) images at the baseline of Case 3 with a type 3 macular neovascularization (MNV) in the left eye. Top row: FA and ICGA of the right eye (A and B) do not show any signs of an MNV. FA of left eye (C) shows leakage in the macula area. ICGA image of the left eye (D) shows hyperfluorescence corresponding to the leakage of FA. Bottom row: OCT image of the right (E) eye appears normal but the left (F) eye shows cystoid macular edema with pigment epithelial detachment (PED). The irregularities of the RPE are due to drusen.

**Fig 2:**
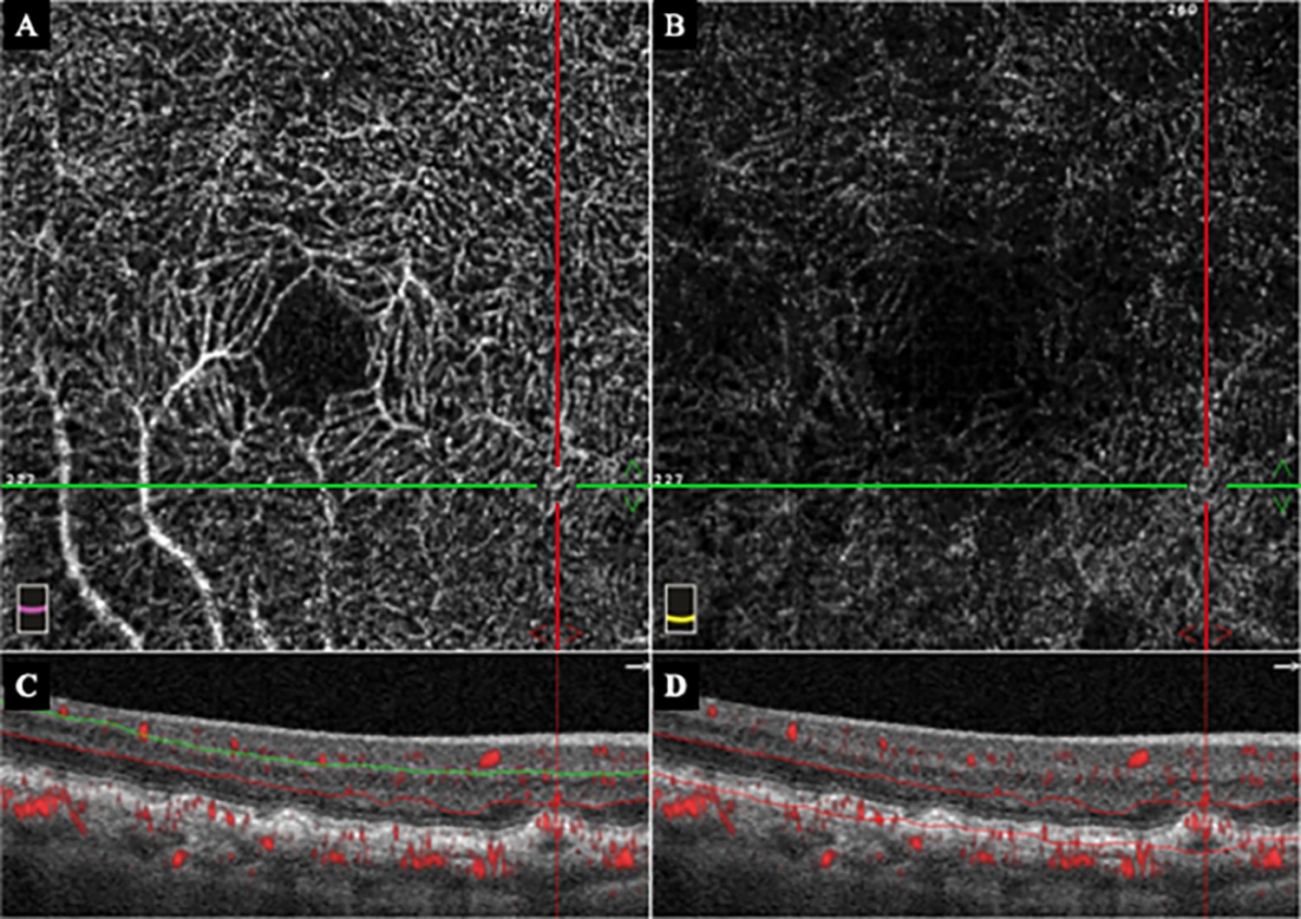
*En face* optical coherence tomography angiographic (OCTA) images of the right eye 3 months prior to the onset of the exudative type 3 MNV. Same case shown in Fig 1. A: Early *en face* OCTA image of the right eye showing deep capillary plexus (DCP) with abnormal blood pattern in the inferior-nasal area of the macula located at the intersection of the horizontal (green) and vertical (red) lines. B: Early *en face* OCTA image of the right eye showing that the outer retina (OR) region is normal. C: B scan OCTA image of the DCP segment overlayed with blood information shows abnormal blood pattern in the DCP segment. D: B scan OCTA image of the OR segment overlayed with blood information shows no abnormalities of the blood flow pattern.

**Fig 3:**
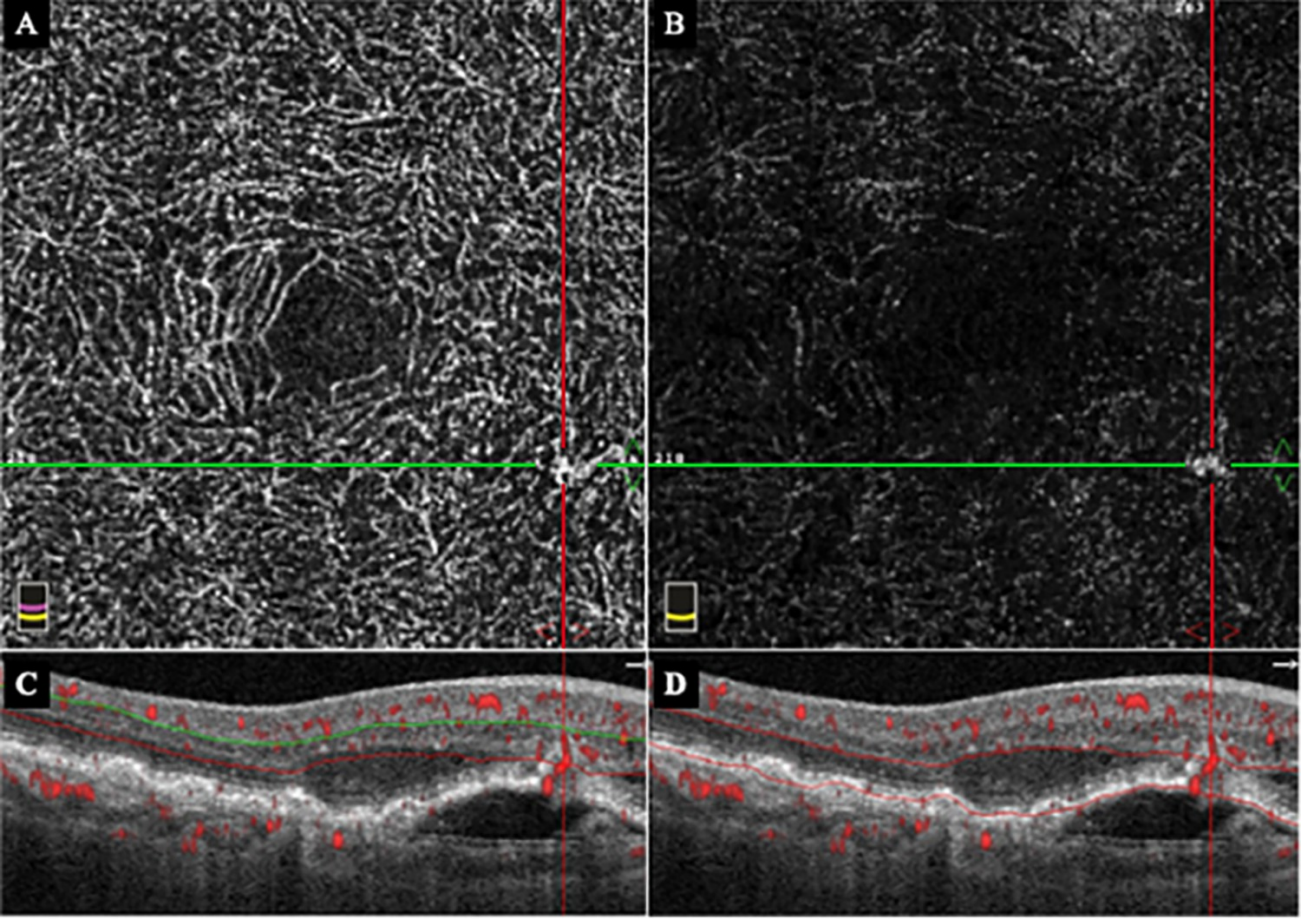
OCTA and OCT B scan images of the DCP and OR segments at the onset of type 3 MNV of the fellow right eye of the same case shown in Fig 1. A: *En face* OCTA image of the DCP of the right eye showing abnormal blood flow pattern in the inferior-nasal area of the macula where the horizontal green and vertical red lines cross. B: *En face* OCTA image of the OR segment showing blood flow pattern of the same area shown in A. C: B scan OCTA image of the DCP segment overlayed with blood information shows the abnormal blood pattern with retinal edema and PED. D: B scan OCTA image of the OR segment overlayed with blood information shows abnormal blood pattern with retinal edema and PED.

A 78-year-old woman was treated for type 3 MNV with a pigment epithelial detachment (PED) and cystoid macular edema (CME) in the left eye (Fig 1). Five months later, a type 3 MNV with PED and CME developed in the inferior-nasal area of the macula of her right eye (Fig 3). OCTA imaging had been done 3 months earlier on this eye, and we examined the OCTA images of the inferior-nasal macula area (Fig 2). We observed abnormal blood flow signals suggestive of non-exudative type 3 MNV without edema only in the DCP (Fig 2A and 2C). OCTA B-scan at the time of the exudation showed that the MNV was tortuous rather than vertically aligned.

Case 5 (Figs 4–6)

**Fig 4:**
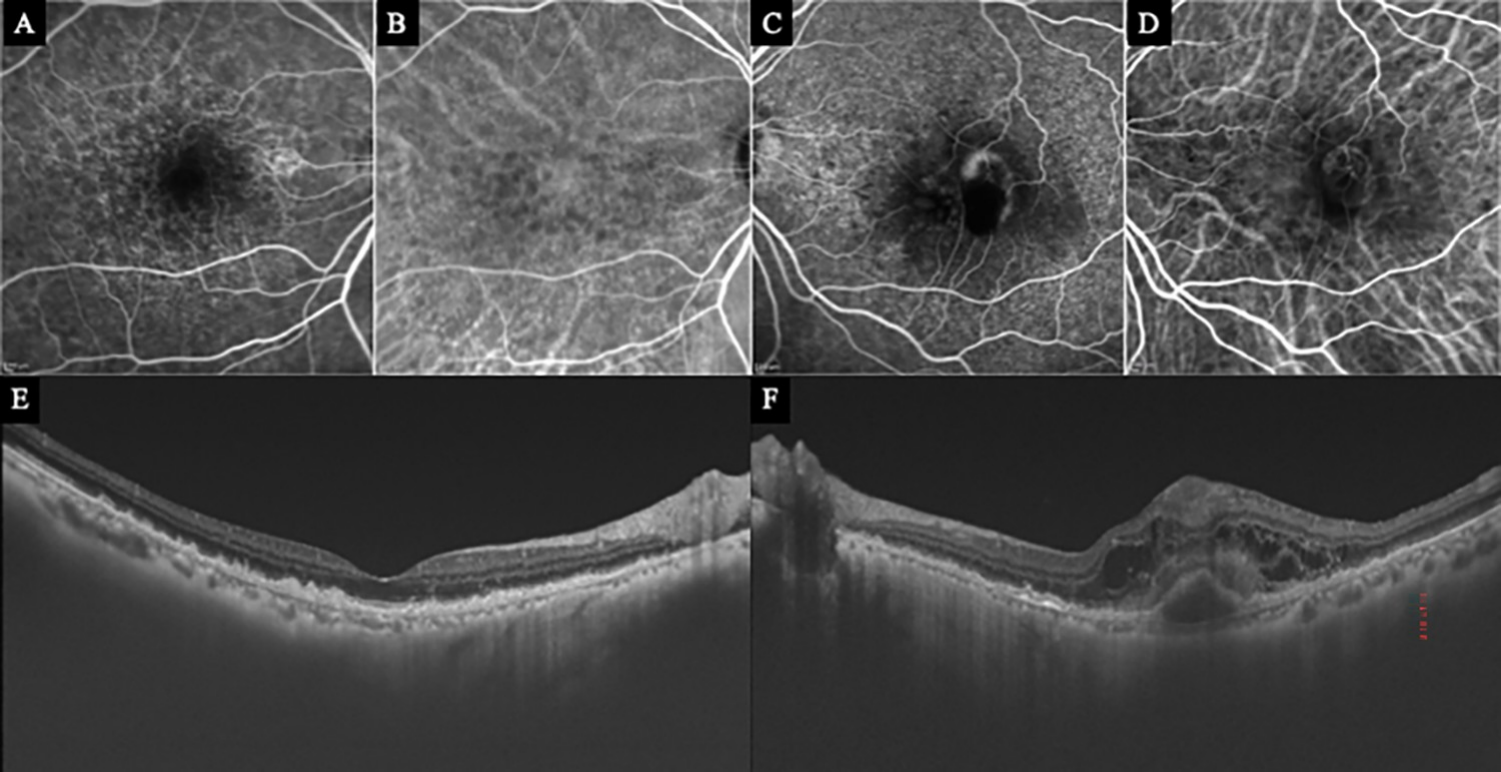
Baseline findings in Case 5 with a type 3 MNV in the left eye. A type 3 MNV developed in the fellow normal right eye during the follow-up period. Top row: FA and ICGA of the right (A and B) show no abnormalities. FA of the left eye (C) shows leakage in the superotemporal region of the fovea. The findings in the temporal fovea could not be confirmed because of blockage by intraretinal hemorrhage. ICGA image of the left eye (D) shows hyperfluorescence as hot spots corresponding to the leakage of FA. Bottom row: OCT image of the right eye (E) has no abnormality other than RPE irregularities due to drusen. OCT image of the left eye (F) shows cystoid macular edema, PED, and intra- and sub-retinal hyperreflective tissue associated with the hemorrhages.

**Fig 5:**
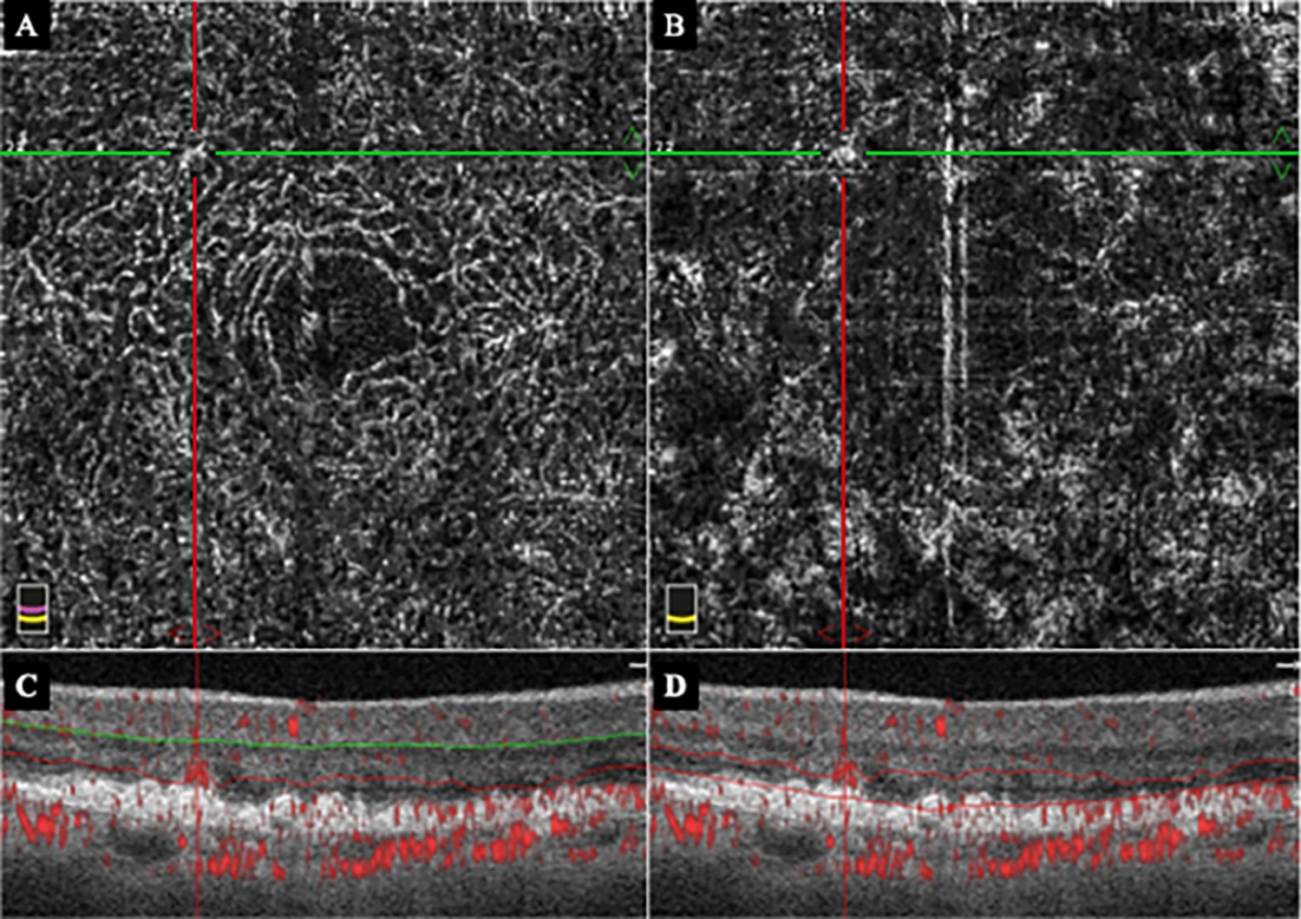
OCTA and OCT images at 3 months before the onset of type 3 MNV of the right eye in the same case shown in Fig 4. A: Prior *en face* OCTA image of the DCP segment showing abnormal blood pattern in the superotemporal area of the macula bordered at the intersection of the horizontal (green) and vertical (red) lines. B: Prior *en face* OCTA image of the OR segment showing abnormal blood pattern in the superior-temporal area of the macula. C: B scan OCTA image of the right eye overlayed with blood information shows the abnormal blood pattern in the DCP segment. D: B scan OCTA image overlayed with blood information also shows the abnormal blood pattern in the OR segment.

**Fig 6:**
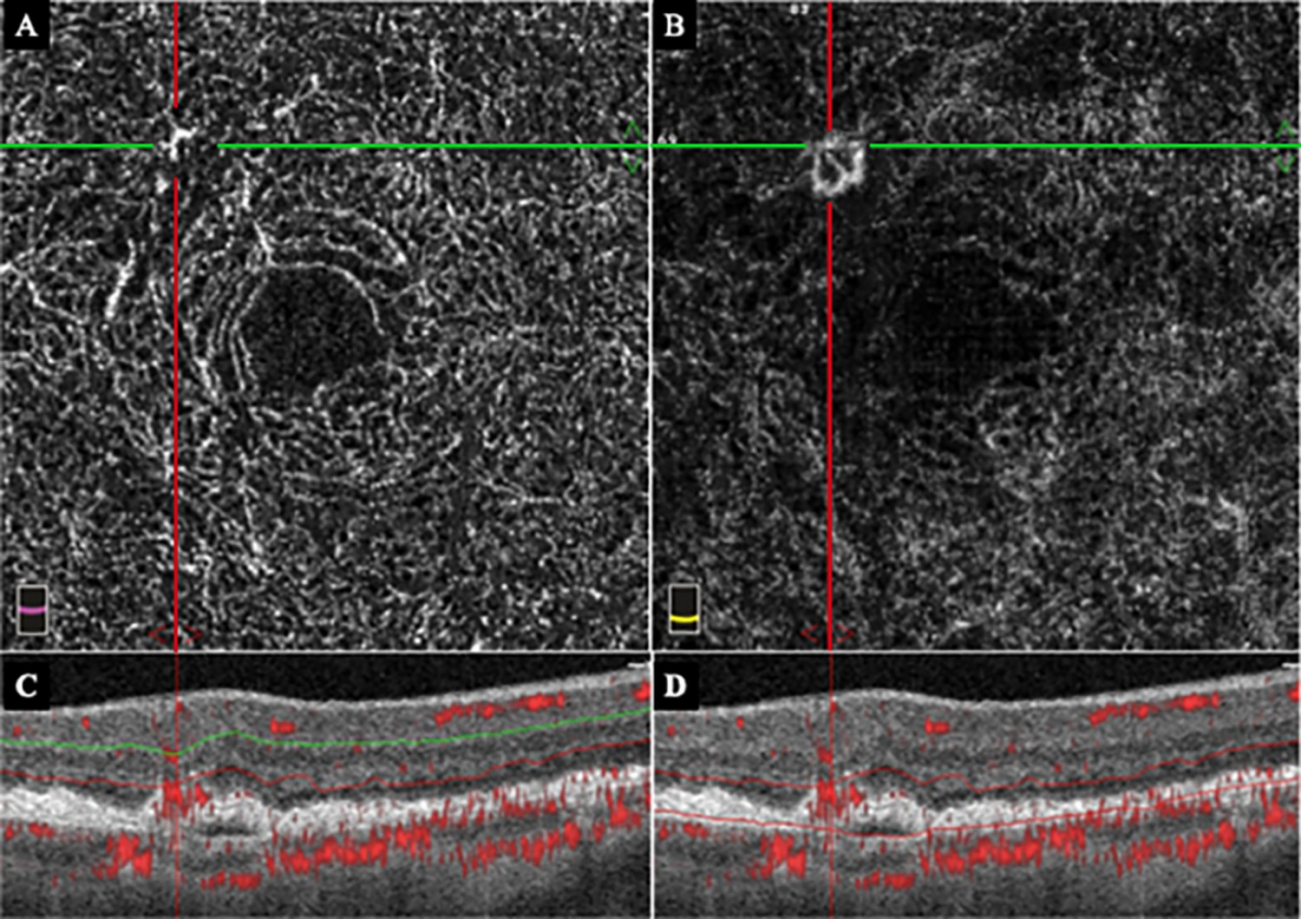
*En face* OCTA and OCT images at onset of the type 3 MNV of the right eye of the same case shown in Fig 4. A: *En face* OCTA image of the DCP segment shows abnormal blood pattern in the superotemporal area of the macula located at the intersection of the horizontal (green) and vertical (red) lines. B: *En face* OCTA image of the OR segment showing abnormal blood patterns in the superotemporal area of the macula located at the intersection of the horizontal (green) and vertical (red) lines. C: B scan OCTA image overlayed with blood information shows the abnormal blood information in the DCP segment. D: B scan OCTA image overlayed with blood information shows the abnormal blood patterns in the OR segments.

A 79-year-old woman began treatment for type 3 MNV with PED and CME in the left eye (Fig 4). One year later, a type 3 MNV with CME developed in the superior-temporal region of the macula of the right eye (Fig 6). Analyses of the OCTA recorded 3 months prior showed abnormal blood flow suggestive of a non-exudative type 3 MNV at the site of the newly developed MNV (Fig 5). These findings were confirmed to be present in the DCP and OR at that time.

## Discussion

During the follow-up of 10 unilateral type 3 MNV cases, OCTA of the fellow normal eyes that developed an MNV showed the blood flow pattern in the DCP and OR. In the 8 eyes with an OCTA recording before the development of exudation of the MNV in the fellow eye, five eyes already had a blood flow pattern suggestive of non-exudative Type 3 MNV. Interestingly, this type of blood flow pattern was detected only at the DCP in three eyes. These findings are important for determining the origin of Type 3 MNVs. In the five eyes in which nonexudative MNVs were observed, the exudation appeared at an average of 3.5 months after the detection of the MNVs in the OCTA images.

Deep retinal vascular complexes were first detected in 1992 by Hartnett et al [1] as "pigment epithelial detachments associated with retinal vascular abnormalities" in eyes with AMD. Since then, various hypotheses have been made regarding the origin of this type of angiogenesis. Yannuzzi et al [2] presented 59 eyes of exudative AMD as stage 1 RAP with neovascularization of retinal rather than choroidal origin. They explained that the intraretinal neovascularization eventually anastomosed with the choroidal neovascularization. However, Gass et al [6] proposed that these neovascularizations originated from the choroid and the retinal vascular reactions followed. They concluded that such neovascularizations were "latent chorioretinal anastomoses". In 2008, Freund et al [3] and Yannuzzi proposed the term type 3 neovascularization to distinguish them from the typical exudative AMD in which the site of neovascularization exists, within the neural retina regardless with or without choroidal neovascularization. Yannuzzi et al [7] also reported that the type 3 MNV may have a sub-RPE origin as well as an intraretinal origin.

The capabilities of OCTA have advanced by the development of new examination methods. Thus, Sacconi et al. [5] analyzed the OCTA findings at the nascent stage of type 3 MNV and reported that there was already hyperreflective material with blood flow information in the DCP or OR before the exudation developed, i.e., the non-exudative type 3 MNV stage. Although the incidence of type 3 MNV is low in Japanese patients[8, 9], Kataoka et al.[10] reported a stage 1 case of type 3 MNV in the OCTA images in a Japanese patient. They showed the presence of blood flow pattern originating from the DCP in the OCTA images. In our study, OCTA at the nascent stage of type 3 MNV showed the blood flow pattern in 5 of the 8 eyes before the exudation occurred. In 3 of these eyes, the blood flow information was observed only in the DCP. These findings suggest that eyes have MNV originating from the DCP at least in some type 3 MNV eyes.

On the other hand, Borrelli et al [11] analyzed the three-dimension OCTA images in type 3 MNV and identified thread-like neovascularizations within the retina. They reported that the location of neovascularization in type 3 MNV was difficult to determine because they were not always present vertically when evaluated three-dimensionally. This meant that the MNVs in the DCP may not be detected in some cases due to image analysis problems. One of our cases had a vertical and slightly tortuous blood vessel pattern (Fig 3).

Our results indicated that abnormal blood flow information was present in the prior OCTA images only after the appearance of exudation. Even clinically, it is difficult to determine whether the blood flow pattern can really be considered an indicator that exudation will occur. Sacconi et al [5] reported a case of spontaneous resolution of abnormal flow signals without the appearance of exudation in one eye. Sacconi et al. [12] also reported that of the 15 unaffected eyes with type 3 MNV, non-exudative MNV in the fellow eyes developed in 5 eyes after an average of 13 months and exudation occurred 8 months later. These results are similar to our findings. The average time to exudation in our cohort was 3.5 months which is slightly shorter but this may be related to the follow-up period. Even if a non-exudative type 3 MNV is confirmed, it does not mean that treatment should be begun. This is a problem that needs to be confirmed in a prospective study in the future.

Although the number of cases was limited, we were able to confirm that the abnormal blood flow information of non-exudative type 3 MNV in OCTA was observed in five eyes before the appearance of exudation even though three of which were identified only in the DCP. This might indicate that the type 3 MNV originates from intraretinal neovascularization in some cases. On the other hand, the detection of non-exudative type 3 MNV is difficult to determine reliably before the exudation appears, and manual in-depth evaluations of the OCT and OCTA images are necessary for the early diagnosis of type 3 MNV. In unilateral Type 3 MNV, it is important to follow the development in the fellow eye, and OCTA is a necessary examination in such cases.
